# Oral Microbiota Dysbiosis in Male HIV Patients: Comparative Analysis of Candidiasis and HPV-Associated Lesions

**DOI:** 10.3390/microorganisms13092121

**Published:** 2025-09-11

**Authors:** Magnolia del Carmen Ramírez-Hernández, Javier Gaytán-Cervantes, Carolina González-Torres, Miguel Ángel Loyola-Cruz, Rebeca Eunice García-Mendiola, Clemente Cruz-Cruz, Iliana Alejandra Cortés-Ortíz, Eduardo García-Moncada, Teresa López-Flores, Emilio Mariano Durán-Manuel, Nancy Gómez-Mancilla, María Fernanda Oviedo-López, Carlos Alberto Jiménez-Zamarripa, Araceli Rojas-Bernabé, Omar Agni García-Hernández, Jonathan Puente-Rivera, Adolfo López-Ornelas, Nayeli Goreti Nieto-Velázquez, Dulce Milagros Razo Blanco-Hernández, Julio César Castañeda-Ortega, Benito Hernández-Castellanos, Gabriela Anaya-Saavedra, Claudia Camelia Calzada-Mendoza, Juan Manuel Bello-López

**Affiliations:** 1 Hospital Juárez de México, Mexico City 07760, Mexico; 2 Sección de Estudios de Posgrado e Investigación, Escuela Superior de Medicina, Instituto Politécnico Nacional, Mexico City 11340, Mexico; 3 División de Desarrollo de la Investigación, Centro Médico Nacional Siglo XXI, Mexico City 06720, Mexico; 4 Clínica Especializada Condesa Iztapalapa, Mexico City 09730, Mexico; 5 Posgrado en Patología y Medicina Bucal, Universidad Autónoma Metropolitana, Mexico City 04960, Mexico; 6 Hospital Psiquiátrico Dr. Samuel Ramírez Moreno, Valle de Chalco Solidaridad 56619, Mexico; 7 Facultad de Medicina, Universidad Nacional Autónoma de México, Mexico City 54090, Mexico; 8 Hospital Nacional Homeopático, Hospitales Federales de Referencia, Mexico City 06800, Mexico; 9 Facultad de Biología, Universidad Veracruzana, Xalapa 91090, Mexico

**Keywords:** HIV, oral microbiota, oral lesions, massive sequencing

## Abstract

Progressive immune damage associated with Human Immunodeficiency Virus (HIV) alters mucosal homeostasis, favouring oral microbial imbalance and the development of opportunistic infections. The aim of this study was to characterize the composition and structure of the oral microbiota in different clinical conditions of HIV infection. A cross-sectional study was conducted in 99 Mexican men divided into five groups: HIV-negative controls, newly diagnosed without antiretroviral treatment, virally suppressed, with oral candidiasis, and with HPV infection. Metagenomic DNA was obtained from salivary samples, and the V1–V3 region of the 16S rRNA gene was massively sequenced. Taxonomic profiles, alpha/beta diversity, differential abundance, microbial co-occurrence networks and degree of dysbiosis were analysed. The results showed distinctive profiles between the groups. Alpha and beta diversity was significantly higher in the groups with oral *Candida* and HPV lesions, reflecting a disturbance of microbial balance. Differential abundance analysis revealed an increase in *Streptococcus*, *Veillonella*, *Lactobacillus* and *Actinomyces* genera in HIV patients, while healthy subjects showed higher abundance of *Neisseria*, *Treponema*, and *Rothia*, associated with a eubiotico state. The group of patients with HPV lesions had the highest number of taxa with differential abundance, suggesting an ecological environment altered by the lesion. Analysis of co-occurrence networks revealed a progressive pattern of microbial complexity: controls presented simple networks with weak positive correlations, while HIV groups showed increased connection density and appearance of structured nuclei. The group of patients with HPV lesions presented the highest connectivity, with multiple strongly correlated cores and core nodes such as *Prevotella melaninogenica* and *Shuttleworthia*. The dysbiosis score increased progressively from healthy subjects to those with HPV lesions, indicating a gradient of oral microbial disruption. These findings suggest that HIV immunosuppression and the presence of oral lesions are associated with enhanced dysbiosis, although their individual contributions could not be independently assessed due to the absence of non-HIV lesion controls. The integration of microbial networks and dysbiosis scores could be useful for assessing mucosal and immune health in people with HIV and used as biomarkers of clinical progression.

## 1. Introduction

Human Immunodeficiency Virus (HIV) infection remains a global public health problem. Despite advances in antiretroviral treatment and the development of new pharmacological strategies, significant challenges remain in its control and prevention [1,2,3]. In Mexico, the recent spike in HIV cases among young people highlights the urgency of reinforcing prevention strategies, as well as expanding access to timely detection and strengthening research on this disease and associated pathologies [4,5,6]. In this context, scientific literature has shown that the progressive immunological deterioration associated with HIV is related to alterations in various mucous membranes and epithelia, which induce favourable conditions for colonization by opportunistic microorganisms and infection by pathogens, with the oral cavity as one of the most exposed [7].

The oral cavity takes on a central role as it constitutes a dynamic and highly regulated microenvironment, whose homeostasis depends on the balanced interaction between the resident microbiota, the host immune system and the integrity of the epithelium [8]. The state of equilibrium between the resident oral microbiota and the immune system is called eubiosis [9]. This balance results in all interactions being in a eubiotic system; however, it can be notably affected by immune dysfunction associated with HIV infection, favouring a transition to a state called dysbiosis [9,10,11]. In people with HIV, this balance can be notably altered, even in the presence of virological control, creating favourable conditions for the development of oral lesions such as candidiasis, human papillomavirus (HPV) types 13 and 32, leukoplakia, or recurrent ulcerative lesions [12,13]. These manifestations not only impact quality of life but may also function as clinical markers of immunosuppression [14,15].

Studies of oral microbiota in a state of dysbiosis have provided insight into the impact of HIV infection on the oral microbiota. Analysis of alpha diversity in people living with HIV has revealed changes in the richness and diversity of the resident microbiota, with an increase in bacterial genera involved in proinflammatory processes such as *Veillonella*, *Prevotella*, *Megasphaera*, and *Campylobacter* compared to seronegative subjects [16,17]. Importantly, even when effective viral suppression has been achieved by antiretroviral therapy (ART), mild to moderate mucosal dysbiosis has been observed to persist, possibly associated with persistent immune disturbances or the indirect effects of ART itself [18,19,20]. This may result in infections by microorganisms of diverse aetiology where fungi of the genus *Candida* and HPV can play an important role as generators of oral lesions [20,21]. This is why the concept of oral dysbiosis studied in various settings, including HIV, has gained relevance, as it is not limited to changes in taxonomic diversity but also involves functional changes, expansion of opportunistic taxa, and modification of interaction networks between microorganisms [21,22,23].

Oral dysbiosis refers to an imbalance in the composition and function of the oral microbiota, characterized by reduced microbial diversity, loss of beneficial commensals, and overgrowth of opportunistic or pathogenic species. In HIV-infected individuals, this imbalance is driven by local immune suppression, altered salivary composition, and chronic inflammation, which persist even under effective antiretroviral therapy (ART). Typical patterns include a decrease in health-associated genera such as *Streptococcus* and *Veillonella* and an increase in potentially pathogenic taxa including *Candida*, *Prevotella*, and *Fusobacterium*, which may contribute to oral lesions and systemic inflammation [10,14,24].

Despite the growing number of studies on oral microbiota in HIV models, there remain gaps in knowledge about its structure and modification at different clinical conditions of infection, particularly in patients with and without ART, and oral clinical manifestations such as candidiasis and HPV infection. There are two main gaps in knowledge regarding oral dysbiosis in HIV: the limited understanding of the specific microbial taxa and community shifts associated with this condition and the scarcity of comparative studies evaluating these microbial changes. This lack of information is particularly relevant in Latin America since, to our knowledge, only Argentina and Brazil have conducted the first studies addressing this issue. In an Argentine study, the oral and anal microbiota of men who have sex with men (MSM) and transgender women with HIV was analysed using massive sequencing (shotgun) [25].

The study identified an increased presence of bacteria associated with periodontal disease, linked to viral load, CD4^+^ count and HPV-related lesions. In the Brazilian work, targeted PCR was used to detect 12 bacterial pathogens, including *Candida* fungi in patients with HIV and periodontitis, finding a high prevalence of these pathogens [26]. Although this work is useful, the intentional molecular approach has limitations, as it does not allow the oral microbiota to be characterized as a whole. Therefore, the need for comprehensive and contextualized research that addresses oral dysbiosis in the Latin American population with a holistic approach, by studying the various clinical conditions of HIV including infectious complications such as oral lesions (candidiasis and HPV-infections), is required. The aim of this work was to characterize the oral microbiota and its associated dysbiosis in Mexican patients at different clinical conditions of HIV, such as candidiasis and HPV infection. Implications of different clinical conditions of HIV as inducers of oral dysbiosis are analysed and discussed.

## 2. Materials and Methods

### 2.1. HIV Population and Controls

This study was cross-sectional, observational and descriptive, and it included a Mexican cohort of HIV-positive (*n* = 76) and HIV-negative (*n* = 23) male participants recruited at Clínica Especializada Condesa Iztapalapa, a medical center for the comprehensive care of sexually transmitted diseases (STDs) located in Mexico City. All participants self-identified as men who have sex with men (MSM). The inclusion of MSM was intentional, aiming to reduce to focus on a group with distinct epidemiological characteristics. While this approach enhances internal validity, we acknowledge that it limits the generalizability of our findings to other populations, including women and men who do not engage in same-sex sexual practices.

Recruited participants were male and over 18 years of age. The HIV-positive participants were classified into two subgroups according to the presence of infectious oral lesions such as oral candidiasis and multifocal epithelial hyperplasia due to HPV, and two other groups consisting of patients with newly diagnosed HIV without ART, and patients with HIV and virological control (with ART). Participants with newly diagnosed HIV were recruited during their first clinical evaluation at Clínica Especializada Condesa Iztapalapa and were immediately referred to the internal HIV care program of the same institution for follow-up, counselling, and ART (Bictegravir/Emtricitabine/Tenofovir Alafenamide-BETA) initiation in accordance with national guidelines. Finally, HPV patients were subjected to surgical treatment, followed by histopathological examination. Nonrecurrences were reported.

Criteria for the clinical diagnosis of oral candidiasis were the following: patchy erythema or red areas usually located on the palate and dorsum of the tongue but occasionally on the buccal mucosa. At times, white spots or plaques of oral candidiasis may also be present. Finally, the observation of hyphae or pseudohyphae on Periodic Acid Schiff (PAS) staining was performed on smears of the lesions. The diagnosis of multifocal epithelial hyperplasia was based on the observation of painless sessile papules on the upper and lower labial mucosa and/or on the lateral edges of the tongue that converged to form plaques with smooth surfaces and was confirmed through histopathological analysis with haematoxylin/eosin (HE).

CD4^+^ cell count/μL was performed using the Pima™ Analyzer (Abbott, Lake Bluff, IL, USA), and viral load (copies/mL) was determined using the m-PIMA™ HIV-1/2 VL Analyzer (Abbott, Lake Bluff, IL, USA). Both tests were performed during the same clinical visit as the oral examination, ensuring that immunovirological data and oral findings were collected simultaneously. Information about ART (if applicable) was collected. The control population (HIV-negative) was recruited from Hospital Juárez de México male volunteers using lateral flow immunochromatography tests, which did not react to HIV IgG antibodies. Additionally, their oral health was assessed through clinical inspection considering complete dentition (no bites), no dentures, no orthodontic treatment and absence of oral or oral candidiasis and multifocal epithelial hyperplasia due to HPV. The characteristics of the study populations are shown in Table 1.

### 2.2. Saliva Sample Collection

Prior to the collection of saliva samples, the absence of dental rinsing for at least eight hours (no dental rinsing) and no consumption of food or beverages were considered. Participants were provided with 10 mL of sterile isotonic saline solution and instructed to thoroughly rinse their mouths using swishing movements for approximately 30 s. The rinse was then expectorated into a sterile 50 mL plastic tube for subsequent oral microbiota analysis. Samples were hermetically sealed, immediately placed on ice and transported to the research laboratory within 2 h for storage at −70 °C until further analysis.

### 2.3. Metagenomic DNA Extraction and Quality Control

Saliva solution samples were centrifuged, and pellets were resuspended in 200 μL of cold isotonic saline solution and incubated with lysozyme solution (20 mg/mL, 20 mM Tris-HCl, pH 8.0; 2 mM EDTA; Triton X-100 (City Chemical LLC, West Haven, CT, USA)) for 2 h at 37 °C. Immediately afterwards, metagenomic DNA was isolated by using the Favorgen^®^ Genomic DNA Kit Commercial (Omega BIO-TEC, Norcross, GA, USA) with minor modifications, including pre-treatment with 20 µL of Proteinase K (20 mg/mL) at 60 °C overnight. Metagenomic DNA samples were quantified by fluorometry using the Qubit 4 Fluorometer (Thermo Fisher Scientific, Waltham, MA, USA). Additionally, the integrity was visualized on horizontal 0.8% agarose gels and was subjected to end-point PCR assays using 27F/1492R primers (V1–V9 regions), to determine if metagenomic DNA samples were amplifiable for the complete *16S rRNA* housekeeping gene (1492 bp) [27].

### 2.4. Ribosomal Libraries Preparation (Hypervariable Regions V1–V3) and Massive Sequencing

The amplicons of the V1–V3 hypervariable regions of the 16S rRNA gene were generated using previously published primers [28], which were ligated to the adapter sequences [29] and used to assemble the DNA libraries. The V1–V3 region of the 16S rRNA gene was selected based on comparative evidence demonstrating improved taxonomic resolution and reduced rates of unclassified sequences in oral microbiome studies when aligned against the Human Oral Microbiome Database [30].

For library assembly, 25 ng of DNA were mixed with 12.5 µL of Go*-Taq* green master mix enzyme (Promega Corporation, Madison, WI, USA) and 10 µM of each primer and amplified using the following conditions: 3 min at 98 °C followed by 25 cycles (20 s at 98 °C, 30 s at 65 °C, 30 s at 70 °C), 5 min at 72 °C and 4 °C hold. Libraries were normalized and sequenced using the 2 × 251 cycle configuration, with 20% Phix control, and 100 µM of the sequencing primers and placed in positions 12, 13, and 14 of the sequencing cartridge. Sequencing of the libraries was performed on the MiSeq platform (Illumina, San Diego, CA, USA).

### 2.5. Quality Control of Sequencing Data

Paired sequencing fastq files were QC inspected for Phred values and for absence of adapters using FastQC v0.11.9 [31]. The data were processed using the DADA2 v1.20.0 pipeline [32]. Standard filter parameters (maxN = 0, truncQ = 8, and maxEE = 2, lengths below 200 bp were discarded) were used. The process of reads continued with dereplication filtering and removal of chimera formation (representing < 2%) using the removeBimeraDenovo option. 16S sequences associated with chloroplasts or mitochondria were removed. The sequences were grouped into Amplicon Sequence Variants (ASVs) with the naive RDP Bayesian classifier of DADA2.

### 2.6. Assignment of Taxonomic Levels and Relative Abundance

Illumina raw sequences were analysed under the QIIME2 command line (version 2021.11) [33]. Following quality control, the processed sequences were separated and analysed by using the SILVA NR99 database (version 138.2), which contains 510,495 sequences, to achieve phylum to species-genus annotation with an identity percentage of ≥99% [34]. For this purpose, the sequences were grouped according to five groups of participants: HIV-negative controls (HP), HIV-positive patients with viral suppression (C_HIV), HIV-positive newly diagnosed without antiretroviral treatment (ND_HIV), HIV-positive with oral candidiasis (Candida_HIV), and HIV-positive with HPV-related oral lesions (HPV_HIV). The taxonomic assignment was conducted by using the R package *phyloseq* (v 1.52.0), allowing classification at five taxonomic levels (phylum, class, order, family, and genus) [35].

### 2.7. Diversity Analysis

Alpha diversity analysis was performed for the five groups of participants (HP, C_HIV, ND_HIV, Candida_HIV, and HPV_HIV) using the *microbiome* and *phyloseq* libraries in R. To assess alpha diversity, the following indices were calculated: Chao1 (total species richness), Gini–Simpson (probability that two randomly selected sequences belong to different species), and the Shannon index (balance between richness and evenness). To assess the degree to which microbial diversity varies between groups, ANOVA test was applied to determine if there were significant differences in the alpha diversity indices performed (*p* < 0.05).

The beta diversity analysis was performed to observe the distribution according to the bacterial composition of the samples based on their similarities. For this purpose, the weighted UniFrac and unweighted UniFrac method coupled to a principal coordinate analysis (PCoA) was used to visualise the variation between microbial communities. Finally, a PERMANOVA analysis was performed to assess whether differences in microbial composition between groups were statistically significant at *p* < 0.05 [36].

### 2.8. Differential Abundance by Study Groups

The differences in taxa between the five groups were studied to identify the ASVs that significantly distinguished each group’s participants. The analyses were done using different models, including Random Forest (RF) using Caret v6.0-94 [37] and MLeval v0.3 package [38] with 1000 trees to build the model, and MicrobiomeAnalyst v2.0 [39]. Cross-validation values were multiplied by 10, and the training set was 90%, while the test set was 10%. In addition, for further validation, a ROC plot and AUC table were determined. Analyses also included a LEfSe test using microbiomeMarker v1.3.3 package [40] with a *q*-value ≤ 0.1 and linear discriminant analysis (LDA score ≥ 2.0).

To analyze the differences in taxa abundance among groups, the Fold Change (FC) and the FDR-adjusted were calculated using DESeq2 [41], and for normalization geometric mean was calculated for each ASV across all samples using TSS. The data were previously filtered based on the significant results of Random Forest and LefSe. Volcano plots were constructed with EnhancedVolcano v1.12.0 [42].

### 2.9. Microbial Association Network Analysis

The correlation network was constructed using NetCoMi v.1.1.0 in Rstudio v.2024.04.2+764 [43]. The netConstruct function was used with the Pearson method as the association measure. To reduce the *network* to a manageable size (sparsification), a cutoff value of 0.3 was applied, and an additional cutoff of 0.3 was added for local false discovery rate correction. Data was normalized with the modified central log-ratio (“mclr”) transformation. The netAnalyze function was implemented using the “cluster_fast_greedy” method. The graphs were generated with the “spring” layout algorithm. Node sizes were adjusted based on betweenness. Estimated associations are shown in red for negative associations and green for positive associations.

### 2.10. Determination of the Degree of Bacterial Dysbiosis

To assess microbial dysbiosis, the dysbiosisR v1.0.4 package in R was used, which allows statistical quantification of microbial imbalance using dissimilarity and community variation metrics [44]. In this study, the dysbiosis score was developed from oral microbiome datasets, based on differences between people living with HIV and healthy controls, and is therefore specific to the oral microbial community. A 5% prevalence filter was applied to include only taxa present in at least 5% of the samples.

The Bray–Curtis distance (method = “bray”) was used as a dissimilarity metric to measure differences between microbial communities based on their relative abundances. To estimate the deviation from the reference state, the function dysbiosisMedianCLV, which calculates the median Community-Level Variation (CLV), was applied. In addition, the Dysbiosis CLOUD Score, based on the distance between each sample and its nearest neighbours within the reference group, was calculated, allowing samples to be categorised as healthy, intermediate or dysbiotic according to their deviation from the normative profile.

## 3. Results

### 3.1. Characteristics of the Study Population

Table 1 presents the characteristics of the five populations included in this study, comprising a total of 99 male participants. Individuals were grouped based on HIV status, virological control and the presence of specific oral lesions (oral candidiasis and HPV infection), including a control group without HIV infection (HP).

The control group (HP), consisting of 23 HIV-negative individuals, had no applicable immunological and virological data, according to rapid immunochromatographic tests. In contrast, the remaining groups consisted of patients living with HIV, differentiated according to their clinical and immunovirological stage. Group C_HIV included 22 patients with confirmed viral suppression (undetectable viral load) and on ART with the BETA regimen. These patients had a mean CD4^+^ cell count of 354 cells/mL (range: 55–671). The ND_HIV group, consisting of 23 individuals with newly diagnosed HIV, was characterized by not having started ART at the time of inclusion in the study. This group showed a mean of 265 CD4^+^ cells/mL (range: 30–749) and detectable viral loads between 2.1 × 10^3^ to 2.5 × 10^5^ copies/mL, reflecting active viral replication. The Candida_HIV group, with 23 participants, consisted of people with HIV who had oral candidiasis, diagnosed clinically and by direct observation of pseudohyphae.

These patients were on ART, with undetectable viral load and an average of 433 CD4^+^ cells/mL (range: 74–893), suggesting a more favourable immune response despite the presence of oral lesions. Finally, the HPV_HIV group, composed of only 8 patients with confirmed oral lesions to HPV infection (multifocal epithelial hyperplasia), showed average CD4^+^ levels of 339 cells/mL (range: 197–639), undetectable viral load and were also on ART. The small size of this group is explained by the low frequency of this condition in the population seen at Clínica Especializada Condesa Iztapalapa.

### 3.2. Quality Control and Error Models in Sequencing Data

The DADA2 analysis for the quality control of the sequencing data of the ribosomal libraries showed that all the reads were of good quality above the *phred* quality score (score 30). Conversely, a parametric error model was run by using the DADA2 algorithm to determine base transitions. Error model constructed was considered adequate, and, therefore, we proceeded to the analysis of taxonomic assignment and relative abundance.

### 3.3. General Characteristics of the Oral Microbiota Profile by Subject and Study Groups

**Figure 1:**
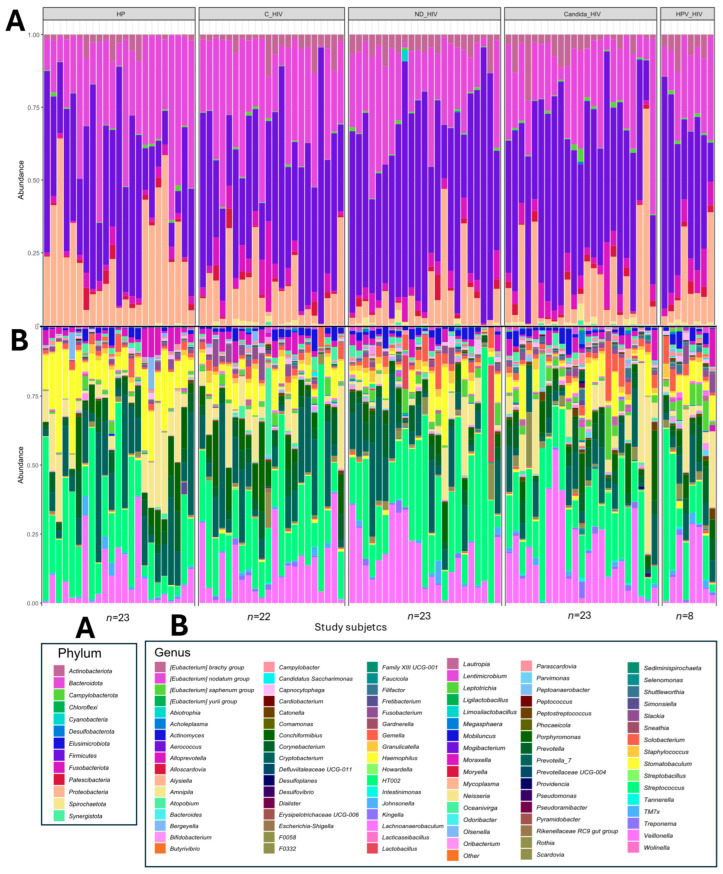
Taxonomic profile and relative abundance of the oral microbiota of the five groups of patients with different clinical conditions of HIV. (**A**). Phila level. (**B**). Genus level. HP: Controls (without HIV), C_HIV: Virally suppressed, ND_HIV: Recently diagnosed HIV, Candida_HIV: HIV and oral candidiasis, HPV_HIV: HIV and HPV-related oral lesions.

Analysis of the taxonomic profile of the oral microbiota of each of the subjects in the five groups with different clinical conditions of HIV revealed the presence of 13 phyla, of which four were predominant (Proteobacteria, Firmicutes, Bacteroidota and Actinobacteriota). In the healthy subjects (HP) group, Actinobacteriota and Firmicutes were the least abundant phyla. In contrast, the C_HIV, ND_HIV and Candida_HIV groups showed a clear predominance of Firmicutes, reaching in some cases almost 100% in relative abundance. Proteobacteria was the predominant phylum in healthy controls, while Patescibacteria were homogeneously distributed in all groups with similar abundances (Figure 1A).

**Figure 2:**
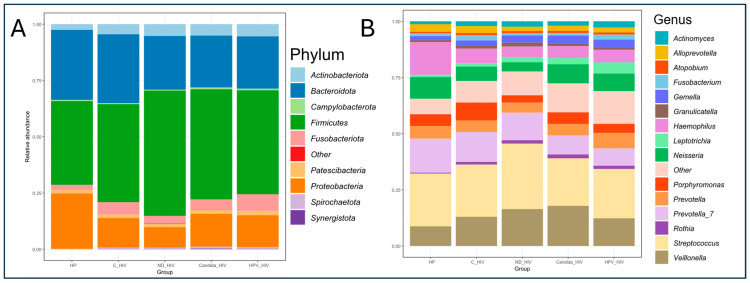
Taxonomic profile and average relative abundance of the oral microbiota of the five groups of patients with different clinical conditions of HIV. (**A**). Phylum level. (**B**). Genus level. HP: Controls (without HIV), C_HIV: Virally suppressed, ND_HIV: Recently diagnosed HIV, Candida_HIV: HIV and oral candidiasis, HPV_HIV: HIV and HPV-related oral lesions.

In the analysis of the taxonomic profile at genus level, an increase in bacterial taxa was observed between subjects and study groups. In healthy control subjects (HP), the oral microbiota was represented, with the genera *Neisseria*, *Haemophilus* and *Rothia* among the most abundant. In contrast, subjects with HIV, especially those with viral control (C_HIV), showed significant abundances of the genus *Corynebacterium*, while those with newly diagnosed (ND_HIV) or with oral candidiasis (Candida_HIV) presented a microbiota dominated by genera such as *Streptococcus* and *Veillonella*, with profiles in some participants almost identical. Finally, in individuals with HPV lesions (HPV_HIV), a greater heterogeneity in the genera identified was observed (Figure 2B). The graphs of the taxonomic composition and relative abundance at the remaining four taxonomic levels are provided in the Supplementary Material.

### 3.4. Taxonomic Diversity and Average Relative Abundance by Participant Groups

The average analysis of the relative abundance and taxonomic composition of the oral microbiota was analysed at the phylum and genus level in five groups of HIV clinical conditions. At the phylum level, of the nine dominant taxa, Firmicutes, Actinobacteriota, Proteobacteria and Bacteroidota were identified as the most abundant with significant variations according to the clinical stage of each group. An increase in Firmicutes was observed in the newly diagnosed HIV group (ND_HIV) compared to the other groups, while Bacteroidota was more abundant in the HP and C_HIV groups. Minority phyla such as Fusobacteriota, Patescibacteria, Actrinobacteriota and Spirochaetota were present in lower proportions in all groups (Figure 2A).

At the genus level, taxonomic diversity analysis revealed a more complex structure of the oral microbiota consisting of fourteen predominant genera. *Streptococcus* and *Veillonella* were the dominant bacterial genera in all groups, with an increase in relative abundance in the group of newly diagnosed participants (ND_HIV) and a slightly smaller increase in the genus *Veillonella* in the group of patients with oral *Candida* lesions (Candida_HIV). In contrast, genera such as *Prevotella* and *Haemophilus* were the most abundant in the healthy control group (HP) and the group with viral control (C_HIV), suggesting a profile associated with oral health. Interestingly, although the genus *Ledtotrichia* was among the least dominant in all groups, a slight gradual increase in abundance was observed in line with the clinical complexity of the study groups. Finally, the genera *Porphyromonas*, *Fusobacterium*, *Atopobium*, *Alloprevotella*, and *Actinomyces* did not show significant changes in abundance in the groups studied (Figure 2B).

### 3.5. Alpha Diversity

**Figure 3:**
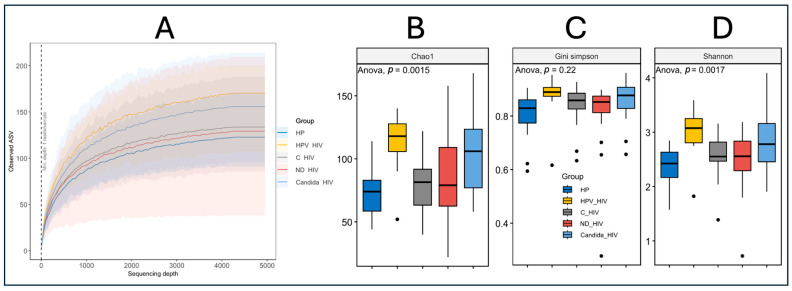
Alpha diversity of the oral microbiota of the five groups of patients with different clinical conditions of HIV. (**A**): Rarefaction curves, (**B**): Chao1 index, (**C**): Gini–Simpson, and (**D**): Shannon. HP: Controls (without HIV), C_HIV: Virally suppressed, ND_HIV: Recently diagnosed HIV, Candida_HIV: HIV and oral candidiasis, HPV_HIV: HIV and HPV-related oral lesions. Statistical significance *p* < 0.05.

Alpha diversity was assessed in the five groups at different clinical conditions of HIV using the Chao1, Gini–Simpson and Shannon diversity indices. For this purpose, a prior analysis of amplicon sequence variant (ASV) richness per study group was performed, showing that all populations reached a plateau, indicating that the sequencing depth was adequate to capture the highest bacterial diversity present, regardless of the numerical disparity of the groups, mainly those participants with oral HPV lesions *(n* = 8) (Figure 3A). Interestingly, the curves corresponding to the HPV_HIV and HP groups showed higher cumulative ASV richness, even though the group of participants with oral HPV lesions was smaller compared to the other clinical conditions. Finally, the remaining three groups (C_HIV, ND_HIV, and Candida_HIV) showed similar behaviour in terms of richness.

Analysis of alpha diversity together with analysis of variance (ANOVA) of the Chao1 index showed significant differences between groups (*p* = 0.0015), with higher species richness in participants with oral HPV (HPV_HIV) and *Candida* (Candida_HIV) lesions, while the control group (HP) had the lowest richness values (Figure 3B). The C_HIV and ND_HIV groups showed intermediate richness values, higher than HP, but lower than HPV_HIV and Candida_HIV, which suggests a moderate impact of HIV infection in the absence of oral lesions. In contrast, the Gini–Simpson index, which assesses equity and diversity by considering both species richness and abundance distribution, did not show statistically significant differences between groups (*p* = 0.22), although values were consistently high (close to 1), suggesting an overall high species diversity (Figure 3C).

Finally, the Shannon index, which integrates both richness and evenness, showed significant differences between groups (*p* = 0.0017), again highlighting the groups of participants with oral lesions (HPV_HIV and Candida_HIV) as having higher microbial diversity, while the control group (HP) maintained lower values, probably reflecting a more conserved and possibly more stable oral microbiota compared to the other groups analysed (Figure 3D).

### 3.6. Beta Diversity

**Figure 4:**
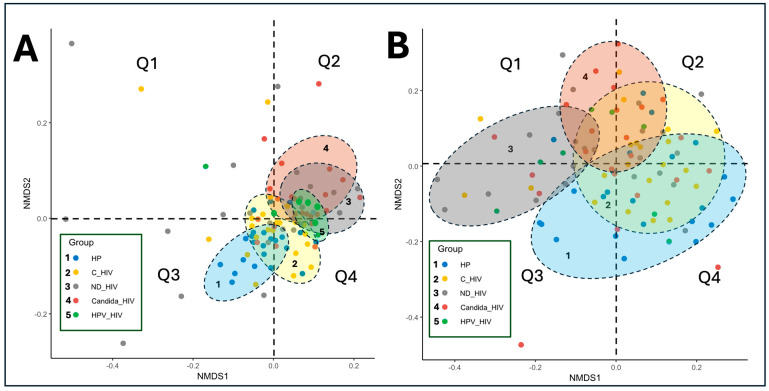
Beta diversity of the oral microbiota of the five groups of patients with different clinical conditions of HIV by PCoA weighted (**A**) and unweighted (**B**) UniFrac. HP: Controls (without HIV), C_HIV: Virally suppressed, ND_HIV: Recently diagnosed HIV, Candida_HIV: HIV and oral candidiasis, HPV_HIV: HIV and D: HPV-related oral lesions.

Beta diversity of the oral microbiota among the five groups of clinical conditions of HIV infection was assessed using non-metric multidimensional scaling (NMDS) analysis from UniFrac distances (weighted and unweighted). Figure 4A, corresponding to the unweighted analysis, shows a partial separation of the composition of all groups. The group of healthy subjects (HP) was mainly distributed in quadrant Q3, while the group of subjects with viral control (C_HIV) and those with oral HPV lesions (HPV_HIV) were clustered in quadrant Q4, showing a relative closeness in their composition.

Conversely, the group of participants with newly diagnosed HIV (ND_HIV) and oral *Candida* lesions (Candida_HIV) presented a larger dispersion, particularly with some participants farther apart in Q2, suggesting greater heterogeneity in microbial composition in these patients. This observation also applies to those patients with newly diagnosed HIV (ND_HIV) and those with viral control (C_HIV).

In Figure 4B, the weighted analysis is shown, where the compositional differences become more evident, reflected by the separation of the taxonomic composition of the oral microbiota of each subject. This phenomenon shows the influence of the wide taxonomic diversity and relative abundance of taxa in each group on this separation. It is observed that the HP and HPV_HIV groups appear to form a single cluster in quadrants Q3 and Q4, while the ND_HIV and Candida_HIV groups cluster predominantly in Q1 and Q2, evidencing similar compositional patterns. In this analysis, the ND_HIV and Candida_HIV groups show the greatest dispersion within their own clusters, which may suggest a less stable oral microbiota. Finally, the group of participants with viral control (C_HIV) show significant overlaps with the Candida_HIV, HPV_HIV and HP groups, suggesting that they share taxonomic compositional characteristics with each other.

### 3.7. Differential Species Abundance Between Groups

**Figure 5:**
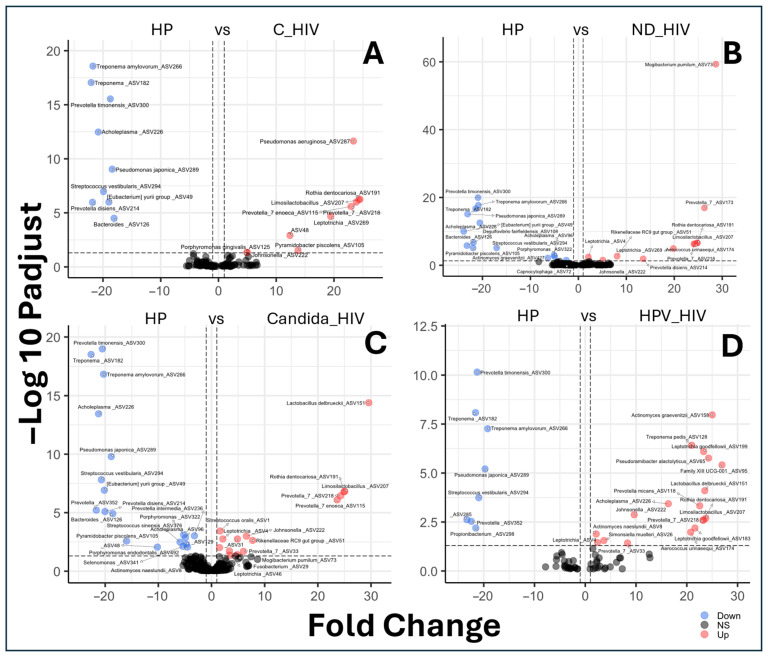
Differential species abundance between groups by biplots analysis of the oral microbiota of the five groups of patients with different clinical conditions of HIV. (**A**): HP vs. C_HIV (Controls vs. Virally suppressed), (**B**): HP vs. ND_HIV (Controls vs. Recently diagnosed HIV), (**C**): HP vs. Candida_HIV (Controls vs. HIV and oral candidiasis), and (**D**): HP vs. HPV_HIV (Controls vs. HIV and HPV-related oral lesions).

The set of volcano plot graphs corresponding to panels A to D shows the comparisons of differential abundance of amplicon sequence variants (ASVs) between the different clinical conditions of HIV and the control group (HP) (Figure 5). The taxa representing the healthy control group (HP) that showed a higher abundance of ASVs and are possibly associated with an oral eubiosis-associated microbiota in our cohort were: *Treponema amylovorum*, *Treponema_ASV182*, *Prevotella timonensis*, *Acholeplasma*, *Pseudomonas japonica*, *Streptococcus vestibularis*, *Eubacterium yurii*, *Prevotella disiens*, *Bacteroides_ASV126*, *Desulfovibrio fairfieldensis*, *Porphyromonas_ASV322*, *Porphyromonas endodontalis*, *Actinomyces graevenitzii*, *Pyramidobacter piscolens*, *Acholeplasma_ASV96*, *Selenomonas_ASV341*, *Propionibacterium*_ASV298, and *Prevotella_ASV352* (Figure 5A–D). In contrast to the group of subjects with viral control (C_HIV), they showed differential abundances represented by eight taxa, notably *Pseudomonas aeruginosa*, *Rothia dentocariosa*, *Limosilactobacillus*, and *Prevotella enoeca* (Figure 5A).

For the group of patients with newly diagnosed HIV (ND_HIV), of the nine taxa identified, *Mogibacterium pumilum*, *Prevotella*, and *Aerococcus urinaeequi* showed the highest differential abundance (Figure 5B). For the Candida_HIV group, 15 taxa were identified, with *Lactobacillus delbrueckii*, *Rothia dentocarinosa*, *Limosilactobacillus*, and *Prevotella enoeca* as the predominant ones (Figure 5C). Interestingly, the group represented by subjects with multifocal epithelial hyperplasia due to HPV (HPV_HIV) showed a differential abundance, represented by seventeen taxa such as *A. actinomyces graevenitzii*, *T. pedis*, *L. goodfellowii*, *Pseudoramibacter alactolyticus*, Family XIII UCG-001, *Lactobacillus delbrueckii*, *Prevotella micans*, *Acholeplasma*, *R. dentocariosa*, *Johnsonella*, *Limosilactobacillus*, *Prevotella*, *A. naeslundii*, *Simonsiella moelleri*, *Leptotrichia*, *Prevotella*, *L. goodfellowii*, and *Aerococcus urinaeequi* (Figure 5D).

### 3.8. Comparative Analysis of Microbial Co-Occurrence Networks

**Figure 6:**
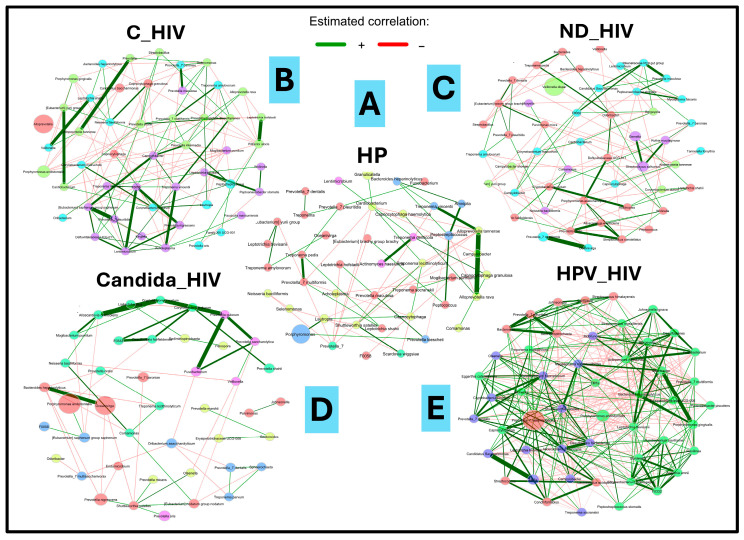
Microbial co-occurrence networks analysis of the oral microbiota of the five groups of patients with different clinical conditions of HIV. (**A**): HP: Controls (without HIV), (**B**): C_HIV: Virally suppressed, (**C**): ND_HIV: Recently diagnosed HIV, (**D**): Candida_HIV: HIV and oral candidiasis, (**E**): HPV_HIV: HIV and HPV-related oral lesions.

Comparison of bacterial co-occurrence networks between the five groups of HIV clinical conditions analysed revealed a progressive transition from a state of equilibrium in healthy individuals (HP) to an imbalance in the context of the underlying pathology (HIV). This was a function of the degree of virological control and the presence of oral *Candida* and HPV lesions. For the purposes of this work, only all interactions were analysed with emphasis on the strong positives in each study group. As can be seen in Figure 6A, in the healthy control group (HP), the network showed a predominance of weak positive correlations; in contrast to the strong interactions, which were in lower proportion. These were represented between the genera *Treponema/Alloprevotella*, *Treponema/Actinomyces*, and *Treponema/Prevotella*. Furthermore, the interaction between the most abundant genus (*Porhyromonas*) was weakly positive with the genus *Lautropia.* Negative interactions were rare compared to the rest of the study groups. In HIV patients under viral suppression (C_HIV), a marked increase in network density and complexity was observed (Figure 6B). Despite virological control, the bacterial community showed moderate connectivity, with a higher number of positive and negative correlations. Although, in this group of patients, *Pseudomonas aeruginosa*_ASV287 was detected as the taxonomic group with the highest differential abundance in the biplot analyses (Figure 5B), it did not particularly show interactions of any kind. In contrast, new strong positive interactions appeared between taxa such as *Veionella/Leptotrichia*, *Cardiobacterium/Eubacterium*, and *Prevotella/Prevotella*, *Leptotrichia/Filifactor*. Interestingly, from this pathological group, strong positive and intermediate interaction cores were detected between more than two genera, represented by commensal anaerobes. An important node for its relative abundance (*Alloprevotella*) shows no positive interactions, and the only negative interaction is with *Peptostreptococcus* (Figure 6B).

In the ND_HIV group, corresponding to individuals recently diagnosed with HIV without ART treatment, the network changes notably, with a decrease in positive interactions and an increase in negative interactions. The appearance of interaction nuclei between new taxa such as *Mycoplasma/Peptoanaerobacter/Prevotella/Rikenellaceae/Candidatus* is observed. The Candida_HIV group, which includes patients with HIV and oral candidiasis, shows a significant reduction in interaction networks. The analysis revealed an isolated core of strong positive interaction between taxa consisting of genera such as *Mogibacterium*, *Alloscardovia*, *Ligilactobacillus*, *Cryptobacterium*, *Corynebacterium*, *Prevotella*, *Fusobacterium*, and *Desulfovibrio.* A single strong positive interaction was identified between *Bacteroides* and *Oceanivirga*, the latter being the one with the highest relative abundance in this interaction network (Figure 6D). Finally, in the HPV_HIV group, corresponding to patients with HIV and oral HPV lesions, the network reaches the highest degree of complexity in all clinical conditions analysed. This complexity is in terms of interaction and density of bacterial taxa. Strong positive increased connectivity is observed, with negative correlations that could be numerically equivalent to each other. Four cores of strong positive communication were identified, the first core is given by *Bacteroides*, *Sediminispirochaeta*, *Johnsonella*, *Centipeda*, and *Streptococcus*; the second important core is given by five taxa (*Chryseobacterium*, *Bordetella*, *Fusobacterium*, *Flexilinea*, *Sneathia* and an unknown taxon named F0332); the third and fourth core, considered the most important, interact strongly with each other through the *Megasphaera* and *Shuttleworthia* taxa. In the third interaction core, 15 main taxa were identified while, for the fourth communication core, nineteen taxa were involved. Of significance is the presence of a dominant node of *Prevotella melaninogenica* in the fourth core that shows simultaneous interaction with several taxa. This complexity of interaction suggests a notable altered microenvironment (Figure 6E).

### 3.9. Determination of the Degree of Bacterial Dysbiosis

**Figure 7:**
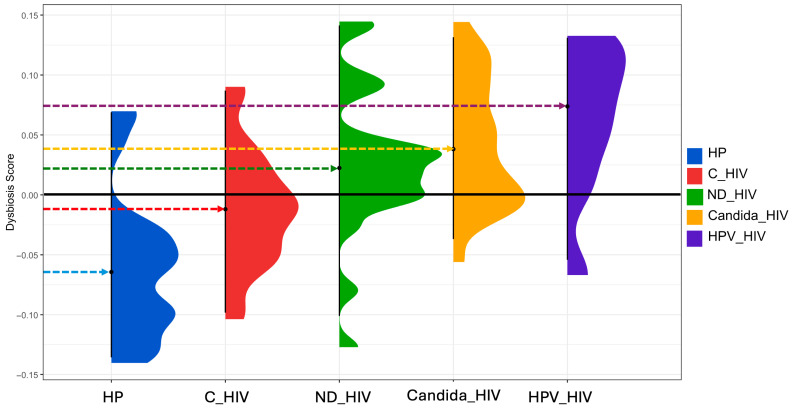
Dysbiosis score of the oral microbiota of the five groups of patients with different clinical conditions of HIV. HP: Controls (without HIV), C_HIV: Virally suppressed, ND_HIV: Recently diagnosed HIV, Candida_HIV: HIV and oral candidiasis, HPV_HIV: HIV and HPV-related oral lesions.

To quantify the microbial imbalance (eubiosis) between the different groups analysed, the *Dysbiosis Score* was used, based on Bray–Curtis dissimilarity metrics and filtering of taxa with a minimum prevalence of 5%. Figure 7 shows the distribution of the dysbiosis score for each of the five study groups, revealing a progressive gradient of bacterial dysbiosis from healthy controls (HP) to patients with HIV and oral *Candida* and HPV lesions. As can be seen, the healthy control group (HP) shows a score that is interpreted as an oral microbiota considered eubiotic, with dysbiosis values centred around zero (−0.06), which establishes an adequate baseline profile.

In contrast, the C_HIV group composed of HIV patients under effective antiretroviral treatment showed a deviation towards positive values (−0.01), suggesting a moderate dysbiosis despite virological control. The ND_HIV group (patients with newly diagnosed HIV) showed a greater heterogeneity in the dysbiosis score, with a median close to zero (0.022), suggesting an unstable microbiota. Patients with oral candidiasis (Candida_HIV) showed marked dysbiosis with a clear shift towards positive values (0.04), indicating a loss of microbial structure possibly attributable to massive *Candida* proliferation and loss of the resident oral microbiota. Finally, the HPV_HIV group had the highest dysbiosis values (0.07), suggesting a pronounced alteration of the oral microbiota. This pattern suggests that the synergism between HIV immunosuppression and the presence of HPV lesions generates a highly symbiotic environment.

## 4. Discussion

HIV infection is characterized by progressive immune dysfunction that alters the homeostatic balance of different host microenvironments, including the oral, intestinal, pulmonary, genital and other microbiota. This dysbiosis can manifest in a variety of ways, from loss or increase in richness and diversity, entry and expansion of opportunistic microorganisms of diverse etiology and alteration of microbial functions, which can induce adverse effects such as systemic inflammation, disease progression and occurrence of co-infections [45,46,47,48]. In the context of the oral microbiota, this community plays a critical role in host defense, regulation of inflammation and prevention of colonization by opportunistic pathogens [8,49,50].

However, under HIV-induced immunosuppression, the oral microbiota may undergo structural collapse, marked by increased richness and diversity and expansion of non-bacterial taxa such as fungi and viruses. This was evident in our alpha diversity analyses, where HPV_HIV and Candida_HIV groups showed the highest species richness, despite the small sample size of HPV_HIV. These findings reinforce the concept of lesion-associated dysbiosis and support the use of alpha and beta diversity metrics in small cohorts. This pattern has been previously reported in HIV-infected pediatric patients with fungal infections, where microbial richness increases alongside reduced similarity of resident microbiota, consistent with displacement dysbiosis [51]. The phenomenon has also been referred to as a “microbial signature” in the literature [52,53]. Beyond disease status, other participant characteristics may influence oral microbiome composition. Although age distribution was comparable between groups and all participants received standardized oral hygiene instructions prior to sampling, individual variability in hygiene adherence and dietary habits could act as confounding factors. These variables were not controlled, but their potential influence could be real.

In addition, key oral health variables such as periodontal status, caries, oral hygiene habits, and smoking were not systematically recorded. These factors are known determinants of oral microbiome composition and may act as uncontrolled confounders. Their absence represents a major limitation of the present study and should be addressed in future research through comprehensive clinical profiling. This behavior could be explained by the fact that the state of chronic inflammation associated with HIV constitutes a niche for increased bacterial diversity and richness, reinforcing the impact of oral lesions and HIV infection on oral dysbiosis. However, there is also literature with opposing results reporting reduced richness and diversity in the oral microbiome among MSM with acute and chronic HIV infection on ART [17]. Importantly, in our study, the population with oral HPV lesions (HPV_HIV), although the smallest (*n* = 8), showed the highest species richness, followed by the population with candidiasis, underscoring the value of alpha- and beta-diversity analyses in small populations.

In this sense, rarefaction curves are essential (Figure 3A), as they allow us to assess whether the sample size has captured the maximum richness of the group in the sequencing assays, providing support to properly interpret the results, including when dealing with populations with low numbers of participants. In terms of beta diversity, it showed distinct clustering between the clinical groups, with discrete separation and overlap between healthy controls and those with virally controlled HIV, reinforcing the idea that while viral control through ART is intended to achieve undetectability and thus halt immune deterioration, oral dysbiosis still exists (Figure 4A,B). This can be seen in Figure 4, where the viral control group (C_HIV) shares compositional characteristics with the other clinical conditions of HIV.

This information confirms that HIV could be induce changes in the composition of the oral microbiota, and that this is exacerbated by the presence of oral lesions. The patterns of separation and overlap in most of the newly diagnosed HIV (ND_HIV) and *Candida* oral lesion (Candida_HIV) groups, as well as more compact clustering between HPV_HIV, C_HIV groups are consistent with that reported by Zhang et al. [54], who identified that oral HPV infection in HIV-positive individuals significantly alters the structure of the oral microbiota, showing differences in beta diversity. Similarly, Noguera-Julian et al. [52] found changes in beta diversity in relation to HIV status, highlighting distinct cluster formation and variability within subgroups of patients with HIV and periodontitis. Our results show that the specific clinical condition of the participants, including the presence of oral lesions and the immune status given by the newly diagnosed HIV, had a major impact on the dispersion of the composition between groups and highlight the role of oral lesions as clinical variables that significantly influence the structure of the oral microbiota. Finally, the fact that in our study the groups with the highest dispersion were precisely those with newly diagnosed HIV (ND_HIV) suggests that this clinical context is associated with a less stable oral microbiota due to the absence of ART and acute damage to the immune system, compared to the other groups that were in a state of undetectability (Table 1).

Differential abundance analysis revealed significantly abundant and typical taxa of the oral microbiota in the group of healthy subjects (HP). It has been reported that the oral microbiota in healthy subjects usually comprises several genera and species including *Treponema*, *Prevotella timonensis*, *Prevotella disiens*, *Streptococcus vestibularis*, *Porphyromonas* (including *Porphyromonas endodontalis*), *Actinomyces graevenitzii*, *Eubacterium*, *Bacteroides*, *Desulfovibrio fairfieldensis*, *Propionibacterium*, *Selenomonas*, *Pyramidobacter piscolens* and *Acholeplasma*, which play important functional roles in oral homeostasis [55,56,57]. For example, *Streptococcus vestibularis* colonises the vestibular mucosa on a regular basis and is involved in maintaining oral pH balance through the production of urease and even hydrogen peroxide [55]. Anaerobic genera such as *Prevotella*, *Porphyromonas*, *Actinomyces*, *Eubacterium*, *Bacteroides*, and *Selenomonas* are frequent components of biofilm and saliva in a state of eubiosis, being a barrier against pathogens [55,57,58,59].

Therefore, their presence in higher abundance in HIV-negative subjects can be interpreted as a representative oral profile of our functionally balanced cohort, as opposed to the dysbiosis observed in the different clinical conditions of this work (Figure 5). In this context, several studies have shown that HIV infection causes significant alterations in the oral microbiota, characterised by an increase in genera such as *Prevotella*, *Rothia*, *Actinomyces*, *Streptococcus* among others compared to healthy individuals [17,60]. *Prevotella* and *Rothia* have been found in abundance in saliva and tongue mucosa samples from people with HIV, correlating with immune response modulation and viral load [17]. These results are consistent with those observed in the present work as these genera and species were abundantly differentiated in the different clinical conditions of HIV infection (Figure 5).

Conversely, species of the genus *Actinomyces*, including *A. graevenitzii* and *A. naeslundii*, are recognised as oral commensals that tend to increase in HIV patients, especially in the presence of immunosuppression or co-infection, and have been associated with opportunistic infections [20]. Increased abundance of these two species was identified in subjects with HPV infection (Figure 5D). Under this last observation, although HIV infection is associated with expansion of certain bacterial taxa, the group with HPV-related oral lesions exhibited a higher differential abundance of 17 taxa. While this may reflect an altered ecological environment, potentially influenced by local epithelial changes or immune modulation, the cross-sectional design limits causal inference [61,62]. These findings should be interpreted as exploratory and may serve as a basis for future longitudinal studies investigating the role of HPV-associated inflammation in shaping oral microbial communities.

Despite ART-mediated viral suppression, mucosal dysbiosis and colonization by opportunistic taxa such as *Pseudomonas aeruginosa* persist, indicating incomplete immune restoration [20,47,63]. *Pseudomonas aeruginosa*, detected in C_HIV patients without co-occurrence interactions (Figure 6B), may reflect exogenous colonization and resistance to local immune mechanisms [63]. These findings highlight that virological control does not equate to full mucosal recovery, and that ecological niches may remain vulnerable to opportunistic pathogens [20]. In the case of the *Rikenellaceae* family found mainly in the digestive tract of warm-blooded animals, it also provides sufficient information to speculate a process of oral dysbiosis in patients with newly diagnosed HIV (ND_HIV) [64]. Taken together, these findings support that the differential abundance profiles observed between groups at different clinical conditions, especially in multifocal epithelial hyperplasia due to HPV, reflect enhanced microbial responses specific to host immune status and co-infections, in this case, to HPV.

To understand the microbial networks that are established in each HIV clinical condition in the groups of patients studied, a co-occurrence analysis was performed that revealed a pattern of increased connectivity and positive correlations especially in those with oral lesions (Figure 6). Comparative analysis between networks of each clinical stage suggests a breakdown in the structure of the “normal” oral ecosystem (Figure 6A), favoring microbial complexity and consequently colonization by opportunistic organisms (Figure 6B–E).

The hyperconnectivity in the networks of Candida_HIV and HPV_HIV populations coincides with patterns described in chronic diseases such as oral cancer [65]. The inclusion of co-occurrence networks was guided by prior richness assessments, specifically rarefaction curves that demonstrated saturation across all groups. This ensured that network construction was based on adequately sampled communities. While these networks do not infer biological function, they offer valuable ecological insights into microbial connectivity and community structure, particularly in the context of oral lesions. Similar ecological approaches have been used to explore microbial community structure and dysbiosis in oral disease contexts [8,23].

Finally, the dysbiosis score allowed us to synthesise our previous findings into a metric that reflected a progressive increase from a state of HP eubiosis towards the clinical stage that has shown the greatest microbiological complexity (HPV_HIV), passing through C_HIV, ND_HIV and Candida_HIV. This index and others have been proposed as a useful biomarker to assess microbiota balance in intervention studies, and its correlation with chronic and infectious diseases [66]. The ability of this metric to identify subtle differences between the various clinical conditions of this work suggests potential utility for monitoring dysbiosis status in people living with HIV, particularly those with oral lesions, where HPV infection became significant due to the microbiological damage it causes (Figure 5 and Figure 6). It is important to highlight that the dysbiosis score applied in this study, based on Shannon diversity and abundance-weighted metrics, is consistent with previously validated approaches in microbiota research. Its application has been documented across conditions such as inflammatory bowel disease, colorectal cancer, and immunodeficiency syndromes, where it has demonstrated utility for clinical stratification [66]. Significant reductions in Shannon diversity have also been reported in contexts such as obesity and inflammatory bowel disease, reinforcing the relevance of diversity-based scores in identifying disease-associated microbial shifts [67,68].

## 5. Conclusions

Our results suggest that HIV infection, the degree of virological control, and the presence of oral lesions are associated with significant alterations in the oral microbiota. Notable changes in bacterial community composition and structure were observed in the presence of oral lesions, suggesting a dysbiosis associated with local immune impairment. These findings add value to the study of the oral microbiome as a microbiological biomarker of immune status and clinical progression in people living with HIV. In this sense, detailed characterization of the oral microbiome under different clinical conditions of HIV infection not only contributes to a better understanding of oral pathophysiology but also opens the possibility of using dysbiosis scores as a prognostic tool. Such scores may prove useful for monitoring clinical response and identifying a return to a eubiotic state as an indicator of improvement. However, their clinical utility remains to be validated in larger, longitudinal cohorts. Finally, although the HPV_HIV group was the smallest cohort, the observed increase in microbial richness, network connectivity, and dysbiosis score suggests a relevant disruption of oral homeostasis. These findings are exploratory and hypothesis-generating, and future studies with larger cohorts are needed to confirm the microbial patterns associated with HPV-related oral lesions.

## Figures and Tables

**Table 1:** Clinical characteristics of the five groups of study.

Code Population	Populations	*n*	HIV Status	Average (Min–Max) CD4^+^ (Cells/mL)	Viral Load (Min–Max) (Copies/mL)	Antiretroviral Therapy
HP	Controls (without HIV)	23	Negative ^a^	Non-applicable	Non-applicable	NA
C_HIV	Virally suppressed	22	Positive	354 (55 to 671)	Undetectable	BETA ^b^
ND_HIV	Recently diagnosed HIV	23	Positive	265 (30 to 749)	2.1 × 10^3^ to 2.5 × 10^5^	Without therapy
Candida_HIV	HIV and oral candidiasis ^c^	23	Positive	433 (74 to 893)	Undetectable	BETA
HPV_HIV	HIV and HPV-related oral lesions ^d^	8	Positive	339 (197 to 639)	Undetectable	BETA
	Total	99				

^a^ By lateral flow immunochromatographic test. ^b^ Bictegravir/Emtricitabine/Tenofovir Alafenamide. ^c^ By clinical characteristics and direct observation pseudohyphae (Pas tinction). ^d^ HPV-related multifocal epithelial hyperplasia.

## Data Availability

The databases of sequences are registered at NCBI with the BioProject ID PRJNA1301429 at http://www.ncbi.nlm.nih.gov/bioproject/1301429 (Accessed Date on 11 August 2025).

## Supplementary Materials

The following supporting information can be downloaded at: https://www.mdpi.com/article/10.3390/microorganisms13092121/s1, Figure S1: Taxonomic profile (Class level) and relative abundance of the oral microbiota of the five groups of patients with different clinical condition of HIV. HP: Controls (without HIV), C_HIV: Virally suppressed, ND_HIV: Recently diagnosed HIV, Candida_HIV: HIV and oral candidiasis, HPV_HIV: HIV and HPV-related oral lesions. Figure S2: Taxonomic profile (Order level) and relative abundance of the oral microbiota of the five groups of patients with different clinical condition of HIV. HP: Controls (without HIV), C_HIV: Virally suppressed, ND_HIV: Recently diagnosed HIV, Candida_HIV: HIV and oral candidiasis, HPV_HIV: HIV and HPV-related oral lesions. Figure S3: Taxonomic profile (Order level) and relative abundance of the oral microbiota of the five groups of patients with different clinical condition of HIV. HP: Controls (without HIV), C_HIV: Virally suppressed, ND_HIV: Recently diagnosed HIV, Candida_HIV: HIV and oral candidiasis, HPV_HIV: HIV and HPV-related oral lesions. Figure S4: Taxonomic profile (Species level) and relative abundance of the oral microbiota of the five groups of patients with different clinical condition of HIV. HP: Controls (without HIV), C_HIV: Virally suppressed, ND_HIV: Recently diagnosed HIV, Candida_HIV: HIV and oral candidiasis, HPV_HIV: HIV and HPV-related oral lesions.

## References

[1] Lindegger D.J. (2024). Advanced Therapies for Human Immunodeficiency Virus. Med. Sci..

[2] GBD 2021 HIV Collaborators (2024). Global, regional, and national burden of HIV/AIDS, 1990–2021, and forecasts to 2050, for 204 countries and territories: The Global Burden of Disease Study 2021. Lancet HIV.

[3] Johnson A.M. (2023). Pandemic HIV and its legacy for medicine and global health. Clin. Med..

[4] Organización Panamericana de la Salud (2025). Lanzan Red de Respuesta al VIH en México. PAHO.

[5] Escandón P. (2025). VIH en México: 202 mil Casos Activos y un Repunte Preocupante en Jóvenes. Excélsior. https://www.excelsior.com.mx/trending/vih-mexico-2025-panorama-general/1722311.

[6] Proceso (2025). Organización VIHVE LIBRE Alerta Sobre Aumento de Casos de VIH. Proceso. https://www.proceso.com.mx/nacional/2025/6/28/organizacion-vihve-libre-alerta-sobre-aumento-de-casos-de-vih-353866.html.

[7] Caputo V., Libera M., Sisti S., Giuliani B., Diotti R.A., Criscuolo E. (2023). The initial interplay between HIV and mucosal innate immunity. Front. Immunol..

[8] Lamont R.J., Koo H., Hajishengallis G. (2018). The oral microbiota: Dynamic communities and host interactions. Nat. Rev. Microbiol..

[9] Huang X., Huang X., Huang Y. (2023). The oral microbiome in autoimmune diseases: Friend or foe?. J. Transl. Med..

[10] Li S., Su B., He Q.S., Wu H., Zhang T. (2021). Alterations in the oral microbiome in HIV infection: Causes, effects and potential interventions. Chin. Med. J..

[11] Fidel P.L., Moyes D., Samaranayake L., Hagensee M.E. (2020). Interplay between oral immunity in HIV and the microbiome. Oral Dis..

[12] Phelan J.A., Abrams W.R., Norman R.G., Li Y., Laverty M., Corby P.M., Nembhard J., Neri D., Barber C.A., Aberg J.A. (2014). Design aspects of a case-control clinical investigation of the effect of HIV on oral and gastrointestinal soluble innate factors and microbes. PLoS ONE.

[13] Rai S., Subramanyam G.B., Kumar G., Bhushan V. (2022). Assessment of oral mucosal lesions among HIV positive transgenders residing in Odisha with and without Antiretroviral therapy. J. Fam. Med. Prim. Care.

[14] Lomelí-Martínez S.M., González-Hernández L.A., Ruiz-Anaya A.J., Lomelí-Martínez M.A., Martínez-Salazar S.Y., Mercado González A.E., Andrade-Villanueva J.F., Varela-Hernández J.J. (2022). Oral Manifestations Associated with HIV/AIDS Patients. Medicina.

[15] Lustosa de Souza B.K., Faé D.S., Lemos C.A.A., Verner F.S., Machado R.A., Ortega R.M., de Aquino S.N. (2023). Associated oral manifestations with HIV southeastern Brazilian patients on antiretroviral therapy. Braz. J. Otorhinolaryngol..

[16] Dang A.T., Cotton S., Sankaran-Walters S., Li C.S., Lee C.Y., Dandekar S., Paster B.J., George M.D. (2012). Evidence of an increased pathogenic footprint in the lingual microbiome of untreated HIV infected patients. BMC Microbiol..

[17] Li S., Zhu J., Su B., Wei H., Chen F., Liu H., Wei J., Yang X., Zhang Q., Xia W. (2021). Alteration in Oral Microbiome Among Men Who Have Sex Whit Men with Acute and Chronic HIV Infection on Antiretroviral Therapy. Front. Cell. Infect. Microbiol..

[18] Lewy T., Hong B.Y., Weiser B., Burger H., Tremain A., Weinstock G., Anastos K., George M.D. (2019). Oral Microbiome in HIV-Infected Women: Shifts in the Abundance of Pathogenic and Beneficial Bacteria Are Associated with Aging, HIV Load, CD4 Count, and Antiretroviral Therapy. AIDS Res. Hum. Retroviruses.

[19] Imahashi M., Ode H., Kobayashi A. (2021). Impact of long-term antiretroviral therapy on gut and oral microbiotas in HIV-1-infected patients. Sci. Rep..

[20] Ramos Peña D.E., Pillet S., Grupioni Lourenço A., Pozzetto B., Bourlet T., Motta A.C.F. (2024). Human immunodeficiency virus and oral microbiota: Mutual influence on the establishment of a viral gingival reservoir in individuals under antiretroviral therapy. Front. Cell. Infect. Microbiol..

[21] Coker M.O., Cairo C., Garzino-Demo A. (2021). HIV-Associated Interactions Between Oral Microbiota and Mucosal Immune Cells: Knowledge Gaps and Future Directions. Front. Immunol..

[22] Li X., Liu Y., Yang X., Li C., Song Z. (2022). The Oral Microbiota: Community Composition, Influencing Factors, Pathogenesis, and Interventions. Front. Microbiol..

[23] Xia Q., Pierson S. (2025). HPV Infection and Oral Microbiota: Interactions and Future Implications. Int. J. Mol. Sci..

[24] Georges F.M., Do N.T., Seleem D. (2022). Oral dysbiosis and systemic diseases. Front. Dent. Med..

[25] Lacunza E., Fink V., Salas M.E. (2023). Oral and anal microbiome from HIV-exposed individuals: Role of host-associated factors in taxa composition and metabolic pathways. npj Biofilms Microbiomes.

[26] Ponce P.N.O., Chaves L.B., Perce-da-Silva D.S., Carneiro-Alencar A.L., Rodolphi C.M., Soares I.F., Rodrigues-da-Silva R.N., Alves-da-Silva A.C., Marques F.V., Peres R.V. (2025). Periodontal Health in Individuals Living with HIV: An Exploratory and Descriptive Molecular Approach of Microbial Interspecific and Intraspecific Diversity in Brazilian Patients. Microorganisms.

[27] DeSantis T.Z., Brodie E.L., Moberg J.P., Zubieta I.X., Piceno Y.M., Andersen G.L. (2007). High-density universal 16S rRNA microarray analysis reveals broader diversity than typical clone library when sampling the environment. Microb. Ecol..

[28] Muyzer G., De Waal E.C., Uitterlinden A. (1993). Profiling of complex microbial populations by denaturing gradient gel electrophoresis analysis of polymerase chain reaction-amplified genes coding for 16S rRNA. Appl. Environ. Microbiol..

[29] Kozich J.J., Westcott S.L., Baxter N.T., Highlander S.K., Schloss P.D. (2013). Development of a dual-index sequencing strategy and curation pipeline for analyzing amplicon sequence data on the MiSeq Illumina sequencing platform. Appl. Environ. Microbiol..

[30] Na H.S., Song Y., Yu Y., Chung J. (2023). Comparative Analysis of Primers Used for 16S rRNA Gene Sequencing in Oral Microbiome Studies. Methods Protoc..

[31] Andrews Simon FASTQC (2020). A Quality Control Tool for High Throughput Sequence Data. https://www.bioinformatics.babraham.ac.uk/projects/fastqc.

[32] Callahan B.J., McMurdie P.J., Rosen M.J., Han A.W., Johnson A.J., Holmes S.P. (2016). DADA2: High-resolution sample inference from Illumina amplicon data. Nat. Methods.

[33] Bolyen E., Rideout J.R., Dillon M.R., Bokulich N.A., Abnet C.C., Al-Ghalith G.A., Alexander H., Alm E.J., Arumugam M., Asnicar F. (2019). Reproducible, interactive, scalable and extensible microbiome data science using QIIME 2. Nat. Biotechnol..

[34] Quast C., Pruesse E., Yilmaz P., Gerken J., Schweer T., Yarza P., Peplies J., Glöckner F.O. (2013). The SILVA ribosomal RNA gene database project: Improved data processing and web-based tools. Nucleic Acids Res..

[35] McMurdie P.J., Holmes P.S. (2013). phyloseq: An R Package for Reproducible Interactive Analysis and Graphics of Microbiome Census Data. PLoS ONE.

[36] Anderson M.J. (2014). Permutational Multivariate Analysis of Variance (PERMANOVA). Wiley StatsRef: Statistics Reference Online.

[37] Caret Package Team (2023). A Short Introduction to the Caret Package. https://cran.r-project.org/web/packages/caret/vignettes/caret.html.

[38] MLeval Package Team (2023). MLeval: Machine Learning Model Evaluation. https://cran.r-project.org/web/packages/MLeval/MLeval.pdf.

[39] Chong J., Liu P., Zhou G., Xia J. (2020). Using microbiomeanalyst for comprehensive statistical, functional, and meta-analysis of microbiome data. Nat. Protoc..

[40] Cao Y., Dong Q., Wang D., Zhang P., Liu Y., Niu C., Marschall T. (2022). MicrobiomeMarker: An R/Bioconductor package for microbiome marker identification and visualization. Bioinformatics.

[41] Love M.I., Huber W., Anders S. (2014). Moderated estimation of fold change and dispersion for RNA-seq data with DESeq2. Genome Biol..

[42] Lewis K.B.S.R. (2022). EnhancedVolcano: Publication-Ready Volcano Plots with Enhanced Colouring and Labeling. GitHub. https://github.com/kevinblighe/EnhancedVolcano.

[43] Peschel S., Müller C.L., von Mutius E., Boulesteix A.-L., Depner M. (2021). NetCoMi: Network construction and comparison for microbiome data in R. Brief. Bioinform..

[44] Shetty S.A., de Steenhuijsen Piters W.A.A. (2022). dysbiosisR: An R Package for Calculating Microbiome Dysbiosis Measures. GitHub Repository. https://github.com/microsud/dysbiosisR.

[45] Anahtar M.N., Byrne E.H., Doherty K.E., Bowman B.A., Yamamoto H.S., Soumillon M., Padavattan N., Ismail N., Moodley A., Sabatini M.E. (2015). Cervicovaginal bacteria are a major modulator of host inflammatory responses in the female genital tract. Immunity..

[46] Lawani M.B., Morris A. (2016). The respiratory microbiome of HIV-infected individuals. Expert Rev. Anti-Infect. Ther..

[47] Pan Z., Wu N., Jin C. (2023). Intestinal Microbiota Dysbiosis Promotes Mucosal Barrier Damage and Immune Injury in HIV-Infected Patients. Can. J. Infect. Dis. Med. Microbiol..

[48] Kehrmann J., Dostmohammadi A., Stumpf A.L., Best L., Consten L., Sievert H., Maischack F., Sammet S., Albayrak-Rena S., Doerr A.K. (2025). Gut microbiota differences linked to weight gain and ART in people living with HIV are enterotype specific and minor compared to the large differences linked to sexual behavior. Front. Cell. Infect. Microbiol..

[49] Wade W.G. (2013). The oral microbiome in health and disease. Pharmacol. Res..

[50] Zhou Y., Gao H., Mihindukulasuriya K.A. (2013). Biogeography of the ecosystems of the healthy human body. Genome Biol..

[51] Mann A.E., O’Connell L.M., Osagie E., Akhigbe P., Obuekwe O., Omoigberale A., Kelly C., DOMHaIN Study Team, Coker M.O., Richards V.P. (2023). Impact of HIV on the Oral Microbiome of Children Living in Sub-Saharan Africa, Determined by Using an *rpoC* Gene Fragment Metataxonomic Approach. Microbiol. Spectr..

[52] Noguera-Julian M., Guillén Y., Peterson J., Reznik D., Harris E.V., Joseph S.J., Rivera J., Kannanganat S., Amara R., Nguyen M.L. (2017). Oral microbiome in HIV-associated periodontitis. Medicine.

[53] Cao P., Zhang Y., Dong G., Wu H., Yang Y., Liu Y. (2022). Clinical Oral Condition Analysis and the Influence of Highly Active Antiretroviral Therapy on Human Salivary Microbial Community Diversity in HIV-Infected/AIDS Patients. Front. Cell. Infect. Microbiol..

[54] Zhang Y., D’Souza G., Fakhry C., Bigelow E.O., Usyk M., Burk R.D., Zhao N. (2022). Oral Human Papillomavirus Associated with Differences in Oral Microbiota Beta Diversity and Microbiota Abundance. J. Infect. Dis..

[55] Dewhirst F.E., Chen T., Izard J. (2010). The human oral microbiome. J. Bacteriol..

[56] Human Microbiome Project Consortium (2012). Structure, function and diversity of the healthy human microbiome. Nature.

[57] Marsh P.D., Zaura E. (2017). Dental biofilm: Ecological interactions in health and disease. J. Clin. Periodontol..

[58] Rajasekaran J.J., Krishnamurthy H.K., Bosco J., Jayaraman V., Krishna K., Wang T., Bei K. (2024). Oral Microbiome: A Review of Its Impact on Oral and Systemic Health. Microorganisms.

[59] Lv C., Li Z., Shi X., Wang Z., Xu Y. (2025). Formation, architecture, and persistence of oral biofilms: Recent scientific discoveries and new strategies for their regulation. Front. Microbiol..

[60] Li Y., Saxena D., Chen Z., Liu G., Abrams W.R., Phelan J.A., Norman R.G., Fisch G.S., Corby P.M., Dewhirst F. (2014). HIV infection and microbial diversity in saliva. J. Clin. Microbiol..

[61] Tezal M., Scannapieco F.A., Wactawski-Wende J., Hyland A., Marshall J.R., Rigual N.R., Stoler D.L. (2012). Local inflammation and human papillomavirus status of head and neck cancers. Arch. Otolaryngol. Head Neck Surg..

[62] Cubie H.A. (2013). Diseases associated with human papillomavirus infection. Virology.

[63] Hegde M.C., Kumar A., Bhat G., Sreedharan S. (2014). Oral Microflora: A Comparative Study in HIV and Normal Patients. Indian J. Otolaryngol. Head Neck Surg..

[64] Dinh D.M., Volpe G.E., Duffalo C., Bhalchandra S., Tai A.K., Kane A.V., Wanke C.A., Ward H.D. (2015). Intestinal microbiota, microbial translocation, and systemic inflammation in chronic HIV infection. J. Infect. Dis..

[65] Nie F., Wang L., Huang Y., Yang P., Gong P., Feng Q., Yang C. (2022). Characteristics of Microbial Distribution in Different Oral Niches of Oral Squamous Cell Carcinoma. Front. Cell. Infect. Microbiol..

[66] Wei S., Bahl M.I., Baunwall S.M.D., Hvas C.L., Licht T.R. (2021). Determining Gut Microbial Dysbiosis: A Review of Applied Indexes for Assessment of Intestinal Microbiota Imbalances. Appl. Environ. Microbiol..

[67] Turnbaugh P.J., Hamady M., Yatsunenko T., Cantarel B.L., Duncan A., Ley R.E., Sogin M.L., Jones W.J., Roe B.A., Affourtit J.P. (2009). A core gut microbiome in obese and lean twins. Nature.

[68] Ott S.J., Musfeldt M., Wenderoth D.F., Hampe J., Brant O., Fölsch U.R., Timmis K.N., Schreiber S. (2004). Reduction in diversity of the colonic mucosa associated bacterial microflora in patients with active inflammatory bowel disease. Gut.

