# Supplementary material for: Oral Microbiota Dysbiosis in Male HIV Patients: Comparative Analysis of Candidiasis and HPV-Associated Lesions
Source: Microorganisms. 2025 Sep 11;13(9):2121. doi: 10.3390/microorganisms13092121 (PMC12473140; doi:10.3390/microorganisms13092121)
Supplement: Supplementary file 1 [file microorganisms-13-02121-s001.zip › microorganisms-3847868-supplementary.pdf]

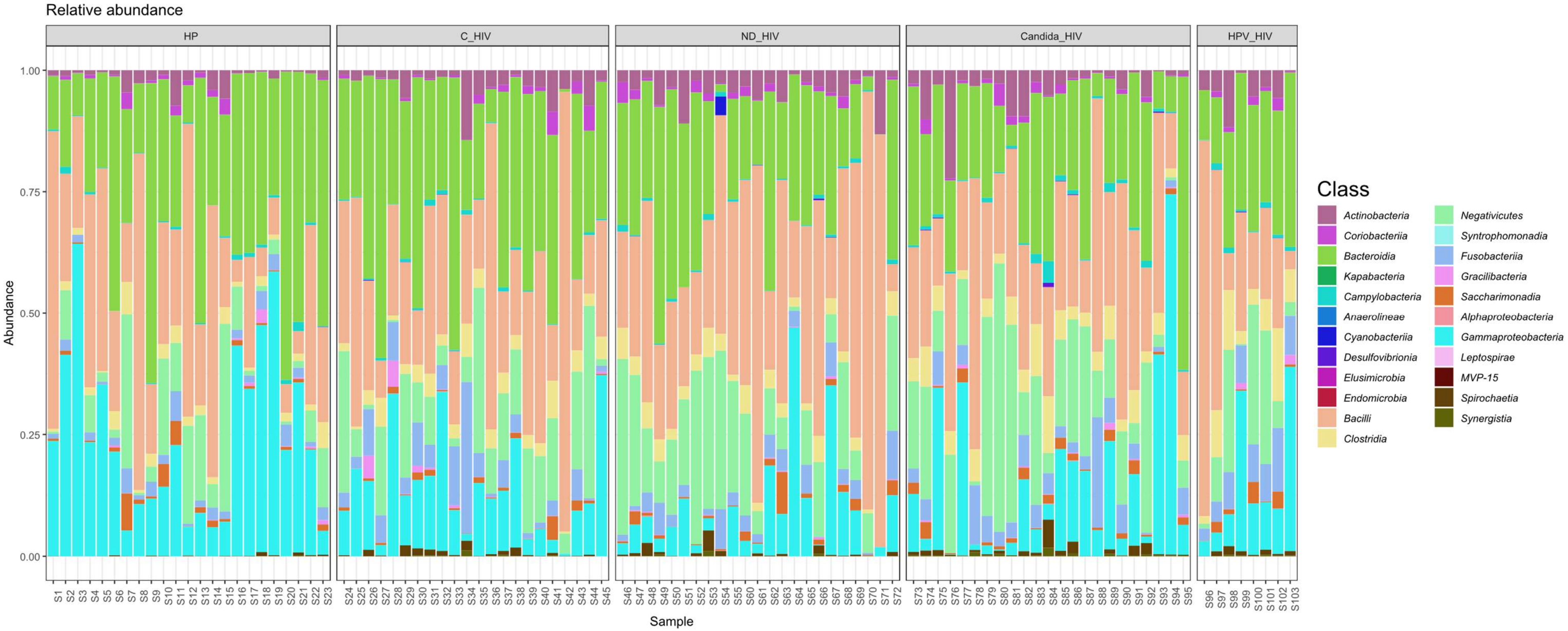


**Supplementary Figure S1:** Taxonomic profile (Class level) and relative abundance of the oral microbiota of the five groups of patients with different clinical condition of HIV. HP: Controls (without HIV), C_HIV: Virally suppressed, ND_HIV: Recently diagnosed HIV, Candida_HIV: HIV and oral candidiasis, HPV_HIV: HIV and HPV-related oral lesions.

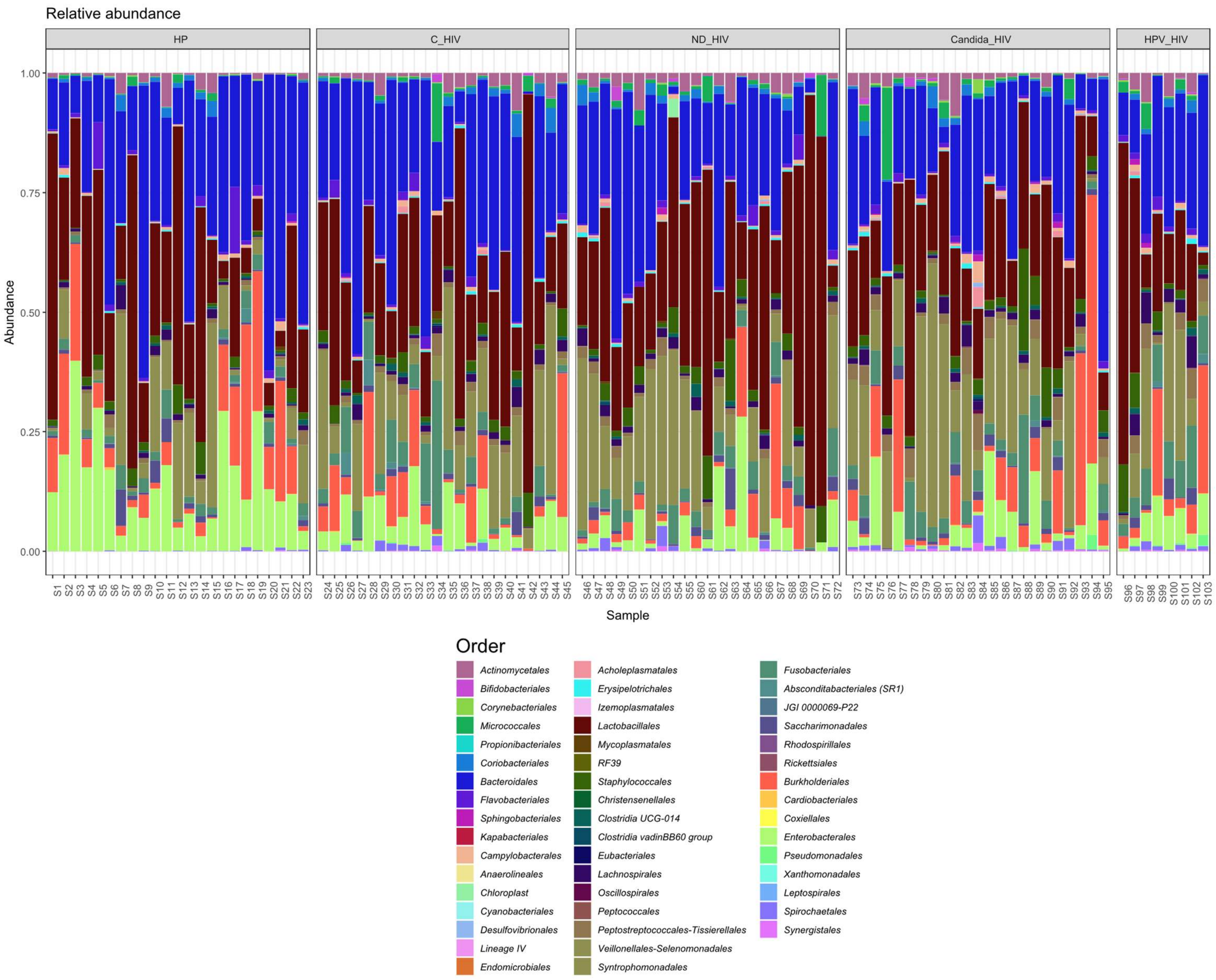


**Supplementary Figure S2:** Taxonomic profile (Order level) and relative abundance of the oral microbiota of the five groups of patients with different clinical condition of HIV. HP: Controls (without HIV), C_HIV: Virally suppressed, ND_HIV: Recently diagnosed HIV, Candida_HIV: HIV and oral candidiasis, HPV_HIV: HIV and HPV-related oral lesions.

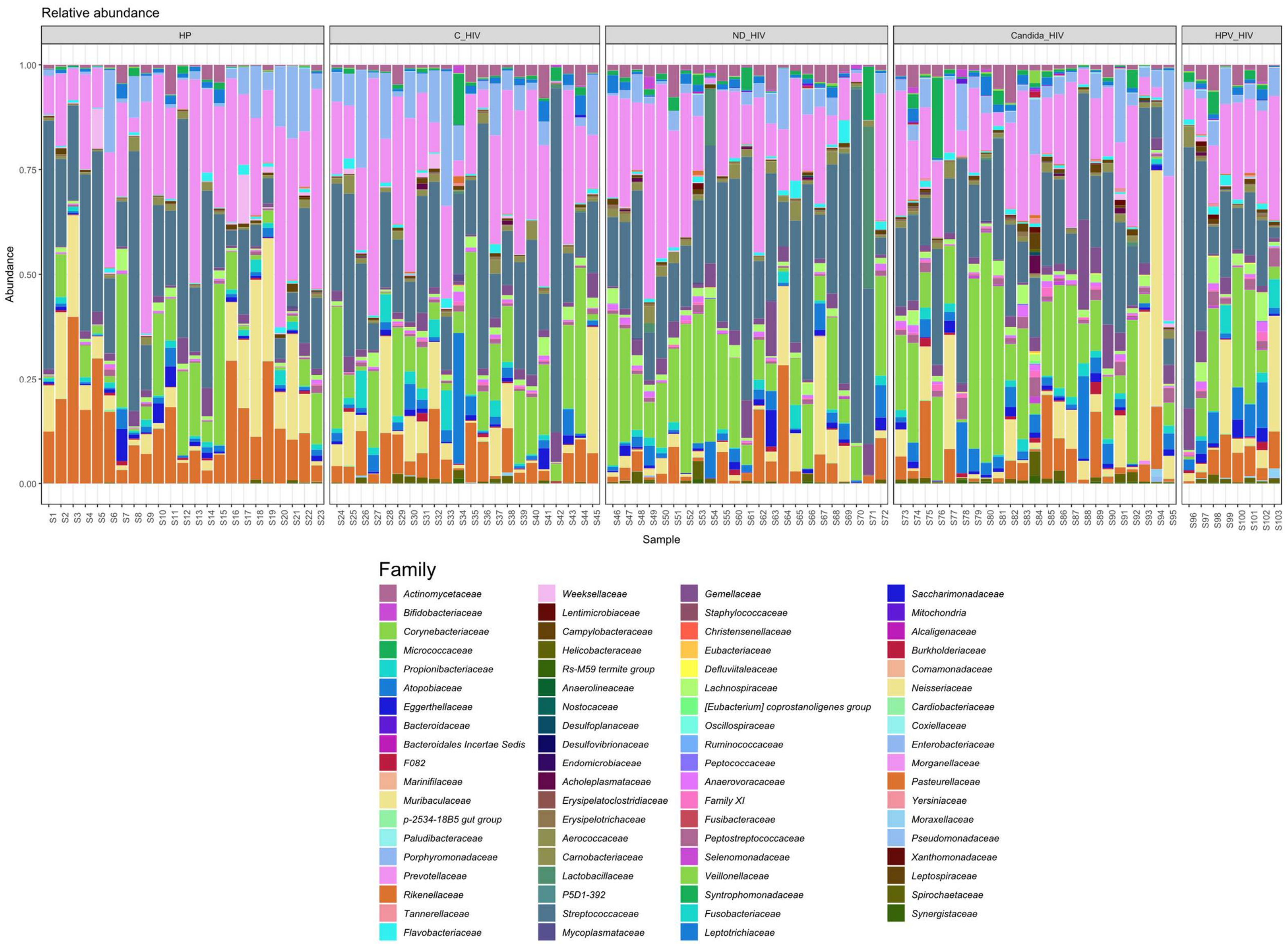


**Supplementary Figure S3:** Taxonomic profile (Family level) and relative abundance of the oral microbiota of the five groups of patients with different clinical condition of HIV. HP: Controls (without HIV), C_HIV: Virally suppressed, ND_HIV: Recently diagnosed HIV, Candida_HIV: HIV and oral candidiasis, HPV_HIV: HIV and HPV-related oral lesions.

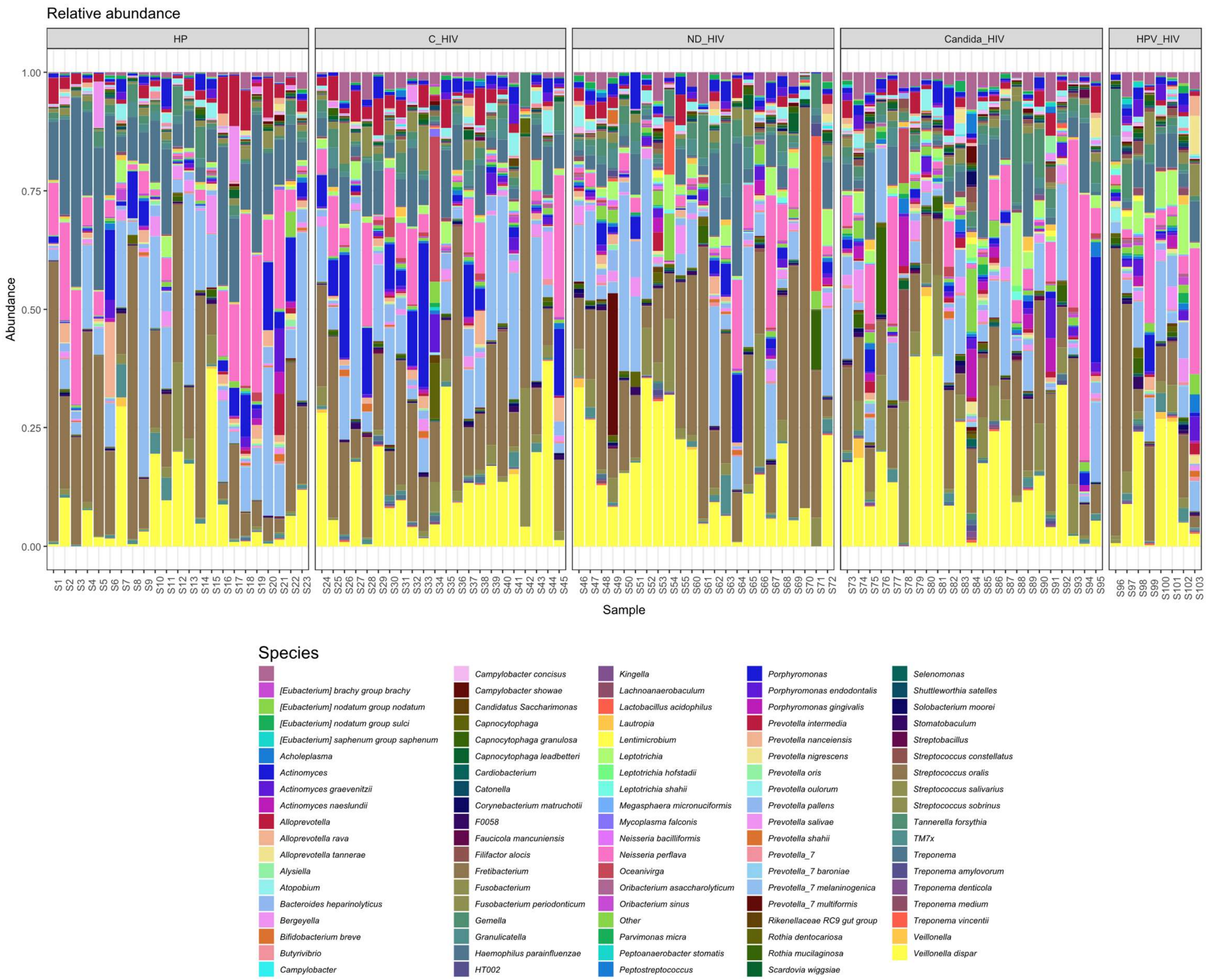


**Supplementary Figure S4:** Taxonomic profile (Species level) and relative abundance of the oral microbiota of the five groups of patients with different clinical condition of HIV. HP: Controls (without HIV), C_HIV: Virally suppressed, ND_HIV: Recently diagnosed HIV, Candida_HIV: HIV and oral candidiasis, HPV_HIV: HIV and HPV-related oral lesions.
